# The relationship between work-family conflict and the level of self-efficacy in female nurses in Alzahra Hospital

**Published:** 2010

**Authors:** Iran Baghban, Marziyeh Malekiha, Maryam Fatehizadeh

**Affiliations:** * Department of Counseling, School of Education and Psychology, The University of Isfahan, Isfahan, Iran; ** Student, Department of Counseling, School of Education and Psychology, The University of Isfahan, Isfahan, Iran

**Keywords:** Conflict (Psychology), self-efficacy, work, family, female, nurses

## Abstract

**BACKGROUND:**

Work-family conflict has many negative outcomes for organization and career and family life of each person. The aim of present study was to determine the relationship between work-family conflict and the level of self-efficacy in female nurses.

**METHODS:**

In this cross-sectional descriptive research, the relationship between work-family conflict and the level of self-efficacy in female nurses of Alzahra Hospital was assessed. Questionnaire, demographic data form, work-family conflict scale and self-efficacy scale were the data collection tools. Content analysis and Cronbach’s alpha were used for evaluating the validity and reliability of questionnaire. The study sample included 160 nurses (80 permanent nurses and 80 contract-based nurses) selected through simple random sampling from nurses working in different wards of Alzahra Hospital. Data analysis was done using SPSS software.

**RESULTS:**

There was significant difference in work-family conflict between the two groups of permanent and contract-based nurses (p = 0.02). Also, a significant difference in the level of self-efficacy was observed between the two groups of nurses (p = 0.03).

**CONCLUSIONS:**

The level of self-efficacy and work-family conflict in contract-based nurses was not acceptable. Therefore, it is suggested to arrange courses to train effective skills in the field of management of work-family conflicts in order to increase the level of self-efficacy for contract-based nurses.

Work and family are central components in people’s lives; much time is spent managing multiple responsibilities.^1^ Work-family conflicts and family-work conflicts are defined as forms of friction in which role pressures from work-family domains are mutually incompatible in some respects.^2^ Work-family conflict is defined as conflict that arises due to work responsibilities interfering with family responsibilities. Similarly, family-work conflict is described as a form of inter-role conflict in which general demands of time are devoted to, and strain created by, the family which interferes with performing work related responsibilities.^3^ Work related tasks encompass hours of paid work and can additionally include overtime work, work related travel and work obligations that are fulfilled at home.^4^ From a work-family perspective, this type of conflict reflects the degree work demands that interfere with family responsibilities.^5^ Family-work conflict occurs when responsibilities associated with one’s family roles interfere with work related demands.^6^ Work-family conflict has significant negative impact on psychological wellbeing and satisfaction.^7^ Much of the research on work-family conflict has focused on several negative outcomes that can occur as a result of this conflict such as a decrease in work-family wellbeing and life and job satisfaction and an increase in occupational burnout or turnover.^8^

In the case of managing the conflict that inevitably arises between personal and career responsibilities assessing work-family conflict,^8^ self-efficacy can provide a unique perspective on what might ultimately help reduce the negative outcomes (e.g., decrease in life and job satisfaction) as a person’s own judgment of the abilities to complete a given task or course of action.^9^ Bandura described the self-efficacy as a key determinate of psychological change, choice of settings and activities, quality of performance in a specific domain and the level of persistence when one meets adverse or negative experience.^10^ These functions of self-efficacy are applicable to work-family conflict. Previous research has linked self-efficacy to multiple-role management.^11^ It is hypothesized that a woman’s level of self-efficacy regarding her work-family responsibilities can help reduce the role conflict and the role overload she may experience.^11^ Erdwins et al examined the relationship of social support, role satisfaction, and self-efficacy to measure the work-family conflict and role overload. Results indicated a negative relationship between work-family conflict and self-efficacy in work and family, suggesting that a woman’s level of work-family conflict decreases as the self-efficacy in her work and family roles increases.^12^ Bernas et al explored self-efficacy as a moderator of the relationship of scholastic aptitude to academic performance and persistence.^13^ The researchers found that self-efficacy for completing educational requirements moderated the relationship between scholastic aptitude and academic performance. Matsui et al examined the level of self-efficacy as a moderator of the relationship between work-family conflict and occupational stress.^14^ Greenhous and Beutell showed that work-family conflict intensifies when work or family roles are salient and central to one’s self-concept.^3^ High level of self-efficacy is very important to manage and moderate work-family conflicts.^15^

This research aimed to find the relationship between work-family conflict and the level of self-efficacy female nurses. The study question was: “Is there difference in work-family conflict and the level of self-efficacy between the two groups of contract-based and permanent female nurses of Alzahra hospital?”

## Methods

In a cross-sectional descriptive study, the relationship between work-family conflict and level of self-efficacy of female nurses was assessed. This study was carried out at Alzahra hospital in Isfahan from June to August 2008. The sample size estimated 180 nurses (80 permanent nurses and 80 contract-based nurses) in the study. These nurses, who were employed in different wards of hospital, were selected by convenience sampling method.

Data gathering was done by means of three questionnaires including individual demographic questionnaire, work-family conflict scale (10 items) by Netmaier in 1996 and self-efficacy scale (24 items) by Cinamon in 2005.^16^ In work-family conflict and self-efficacy questionnaire, the answers were based on Likert scale scored from 1-5. In work-family conflict questionnaire, the total score could range from 10-50. In self-efficacy questionnaire, the total score could range from 24-120. Content analysis and Cronbach’s alpha were used for evaluation of validity and reliability. The reliability of work-family conflict scale was evaluated using Cronbach which was 0.92 for one section and 0.83 for two sections. To assess the reliability of self-efficacy questionnaire, Cronbach’s alpha was also calculated which was 0.80 and 0.91 for one section and two sections, respectively. To assess content validity, two questionnaires (work-family conflict and self-efficacy) were sent to lecturers of the Psychology and Educational school and were edited according to their comments.^16^

## Results

The sample had a mean age of 46 (5.8) years for permanent nurses and 33 (4.4) years for contract-based nurses. Mean record of service was 14 (7.3) years for permanent nurses and 9 (3.1) years for contract-based nurses. The sample had a mean term of marriage of 17 (4.1) years for permanent nurses and 14 (3.3) years for contract-based nurses. Mean age of their husbands was 41 (6.2) years for permanent nurses and 35 (4.1) years for contract-based nurses. Mean age of their children was 7 (3.2) years for permanent nurses and 2 (1.2) years for contract-based nurses. Mean number of children was 8 (3.51) for permanent nurses and 3 (2.41) for contract-based nurses.

Table 1 shows the results of comparing the work-family conflict and self-efficacy between the two groups of permanent and contract-based nurses by t-test. Findings showed a significant difference in the field of work-family conflict between the two groups of nurses (p = 0.02). Also, the results showed a significant difference in the level of self-efficacy between the two groups of nurses (p = 0.03).

**Table 1:** The results of comparing the work-family conflict and self-efficacy between the two groups of nurses (t-test)

Variable	Engagement of situation	Average	Standard Deviation	T	df	P
	Permanent	44.14	33.21			
Work-family conflict	Contract-based	34.07	24.44	0.19	58	0.02
	Permanent	41.24	20.2			
Self-efficacy	Contract-based	22.57	11.51	0.65	41	0.03

## Discussion

The results showed a meaningful difference in the field of work-family conflict between the two groups of permanent and contract-based nurses. The result was probably that the work hours for permanent and contract-based nurses are different; i.e., work hours for permanent nurses are fixed and regular but work hours of contract-based nurses are variable and irregular. Contract-based nurses (with variable work hours and shifting work) manage work-family conflict less successfully than permanent nurses. Carlson et al found that work hours and other environment variables are bigger factors in predicting work-family conflict and role ambiguity.^6^ It has been shown that employees who have high work hours (more than 7 hours per day) perceive more work-family conflict than those who have less work hours (less than 7 hours per day).^17^ It means, there is a direct relationship between work-family conflict and work hours. Edwards et al showed that a negative relationship between flexible and low work hours and work-family conflict in female nurses.^18^ It can be concluded that having a flexible work hours is ranked as the most valuable benefit option by employees. Flexible and low work hours allow employees to adapt their works in a vantage ways to meet their needs.

Also, work-family conflict has significant implications for nurses in terms of profession itself. Work-family conflict was associated with lower job satisfaction, fatigue, emotional distress and depressive symptoms. It can be concluded that work-family conflict has the potential to undermine nurses’ stability to provide high quality care. Moreover, there was significant difference between the two groups of nurses in the level of self-efficacy.^17^ Another study on self-efficacy and three variables of work security, work hours and organizational commitment showed significant relation between work-family conflict and work security and work hours. Also, there was no significant relation between work-family conflict and organizational commitment.^17^

A meaningful relationship between the type of employment and the level of self-efficacy was found. In other words, employees with work security have high level of self-efficacy compared to employees who does not have work security.^17^ Negative relationships between high work-family conflicts and low level of self-efficacy and the importance of self-efficacy in moderating work-family conflicts have been demonstrated.^17^ Women with high level of self-efficacy have experienced less work-family conflict. Nurses with high levels of self-efficacy are more guided by their own internal causes for career than nurses with low levels of self-efficacy. Nurses with lower levels of self-efficacy may be more easily influenced by the external factors.^19^

This research had some limitations. First, although we did not inform nurses about the aim of the study, the attendance of researchers in the wards simulated nurses’ curiosity. We suggest other studies to be conducted with better research methods and larger sample sizes. In addition, there are needs for further studies regarding the correlation of work-family conflict and other personality characters of nurses.

## Conclusion

With regard to significant difference in the work-family conflict and the level of self-efficacy between the two groups of permanent and contract-based nurses, it seems necessary to provide educational courses on management of work-family conflicts and using various techniques to increase the level of self-efficacy for contract-based nurses. Time-management skills, social support and other related skills for decreasing work-family conflicts and training the source of self-efficacy for increasing the level of self-efficacy should be accentuated.

The authors declare no conflict of interest in this study.
